# Health Impact Assessment for Second-Hand Smoke Exposure in Germany—Quantifying Estimates for Ischaemic Heart Diseases, COPD, and Stroke

**DOI:** 10.3390/ijerph13020198

**Published:** 2016-02-05

**Authors:** Florian Fischer, Alexander Kraemer

**Affiliations:** Department of Public Health Medicine, School of Public Health, Bielefeld University, 33615 Bielefeld, Germany; alexander.kraemer@uni-bielefeld.de

**Keywords:** Health Impact Assessment, second-hand smoke, environmental epidemiology, ischaemic heart diseases, COPD, stroke

## Abstract

Evidence of the adverse health effects attributable to second-hand smoke (SHS) exposure is available. This study aims to quantify the impact of SHS exposure on ischaemic heart diseases (IHD), chronic obstructive pulmonary diseases (COPD), and stroke in Germany. Therefore, this study estimated and forecasted the morbidity for the three outcomes in the German population. Furthermore, a health impact assessment was performed using DYNAMO-HIA, which is a generic software tool applying a Markov model. Overall 687,254 IHD cases, 231,973 COPD cases, and 288,015 stroke cases were estimated to be attributable to SHS exposure in Germany for 2014. Under the assumption that the population prevalence of these diseases and the prevalence of SHS exposure remain constant, the total number of cases will increase due to demographic aging. Assuming a total eradication of SHS exposure beginning in 2014 leads to an estimated reduction of 50% in cases, compared to the reference scenario in 2040 for all three diseases. The results highlight the relevance of SHS exposure because it affects several chronic disease conditions and has a major impact on the population’s health. Therefore, public health campaigns to protect non-smokers are urgently needed.

## 1. Introduction

Diseases associated with tobacco use pose a significant burden on individuals, societies, and healthcare systems. Smoking affects not only active smokers but also those who are exposed to second-hand smoke (SHS) in the vicinity of a smoker [1]. Globally, 33% of male non-smokers, 35% of female non-smokers, and 40% of children are regularly exposed to SHS [2]. The ubiquity of tobacco smoke at home or in private establishments, workplaces, and public areas (indoor and outdoor) makes SHS exposure nearly unavoidable [3]. Indoor exposure in particular is highly relevant because people spend 80%–90% of their time indoors; this is especially true for the elderly [4], who develop chronic conditions more frequently than younger people. SHS exposure may lead to several chronic conditions, which are highly relevant in terms of morbidity and mortality for a population’s health [5]. Due to the adverse health effects attributable to several highly prevalent and predominantly chronic diseases, SHS exposure is considered to be the third most frequent risk factor for avoidable deaths [6].

Despite declines in smoking rates in developed countries, exposure to SHS continues to be a widespread health risk and an important public health issue. Studies from Spain [7] and New Zealand [8] have estimated that the number of deaths attributable to SHS exposure among the total population was about 8 per 10,000 deaths. Deaths from SHS exposure in the USA represented a loss of nearly 600,000 Years of Potential Life Lost (YPLL), or an average of 14.2 years per death attributable to SHS exposure. As a result of deaths attributable to SHS, $6.6 billion were lost in productivity [9]. These estimations highlight the relevance of suitable policies to protect non-smokers. Since involuntary SHS exposure is a common and serious public health hazard, appropriate regulatory policies need to be adopted and enforced to prevent adverse health effects [10].

SHS is considered to be the most important environmental risk factor in the etiology of cardiovascular diseases [11]. Therefore, major parts of morbidity and mortality caused by SHS exposure comes from diseases of the cardiovascular system, especially ischaemic heart disease (IHD) [12,13,14]. Respiratory system diseases are another outcome that is causally attributable to active smoking but may also be affected by SHS exposure. Chronic obstructive pulmonary disease (COPD) is a leading cause of premature mortality and morbidity both in developed and developing countries [15]. The International Agency for Research on Cancer (IARC) showed a strong causal effect between SHS exposure and chronic respiratory symptoms in adults [16].

A meta-analysis of 20 studies indicated a strong dose-dependent association between SHS exposure and stroke [17]. Based on data from the German national health survey 1998, it was estimated that 0.9% of strokes among men and 1.5% among women are attributable to SHS exposure. If these data are transferred in absolute numbers, 1873 incident strokes and 770 stroke deaths are assumed to be attributable to SHS exposure every year in Germany. Incident events were predominantly (69.7%) in the age group ≥65 years [18].

High prevalences of SHS exposure as well as adverse health effects attributable to SHS exposure indicate the relevance of smoking bans. The publication of the Surgeon General’s Report in 1986 [19], in which SHS exposure was declared to be a cause of lung cancer in healthy non-smokers, led to an increase in the number of smoking bans and restrictions. A Cochrane review summarizing 25 studies observes consistent positive health effects after the implementation of legislative smoking bans. All studies showed reductions in the duration of self-reported SHS exposure, ranging from 71% to 100%, or in the percentage of those exposed, ranging from 22% to 85% [5]. In Germany, the implementation of smoke-free legislation differs between the federal states [20].

This study aims to quantify the impact of SHS exposure on a population’s health. Therefore, this study will estimate the morbidity for the three outcomes IHD, COPD, and stroke in the German population in 2014 and projected until 2040. These disease entities were chosen, because they impact the health of elderly people in particular. Due to the demographic change, these disease estimates are of major importance. The prediction of the effects of SHS exposure and policies for the protection of non-smokers will be presented in the form of a Health Impact Assessment (HIA).

## 2. Materials and Methods

For the HIA a software tool called DYNAMO-HIA (DYNAmic MOdeling for Health Impact Assessment) was used [21]. DYNAMO-HIA is a generic software tool applying a Markov model, which allows the quantification of the impact of risk factors on health and changes in risk factors due to interventions on various diseases on overall population health [22,23]. DYNAMO-HIA already contains data on some risk factors (smoking, BMI, alcohol), nine diseases (incidence, prevalence, excess mortality), and population data for several countries.

### 2.1. Data Input for Health Impact Assessment

The data for the population living in Germany and for the disease prevalence of the three outcomes—IHD, COPD, and stroke—which are already included in DYNAMO-HIA, were used for the simulation [23]. The effect sizes for the association between SHS exposure and the three outcomes (IHD, COPD, and stroke) were taken from a meta-analysis which was conducted for this HIA. The methods and results of this meta-analysis are described elsewhere [24]. The relative risk (RR) calculated in the meta-analysis are based on studies (cohort and case-control studies) assessing the effect of regular SHS exposure (e.g., spousal smoking or exposure to 20 cigarettes per day). In Table 1 the RR and, in addition, the 95% CI are shown, although only the point estimates were used in the HIA.

**Table 1:** Relative risks for the association between SHS exposure and adverse health outcomes ^1^.

Outcome	RR (95% CI)	
	Men	Women
Ischaemic heart disease	1.06 (0.96–1.19)	1.50 (1.31–1.72)
COPD	1.50 (0.96–2.28)	2.17 (1.48–3.18)
Stroke	1.40 (1.09–1.81)	1.43 (1.28–1.61)

^1^ Data based on a meta-analysis [24].

The RR for each outcome, stratified by sex, was used in the simulation. Furthermore, the prevalence of SHS exposure was included in the analysis based on data from the GEDA survey (*Gesundheit in Deutschland Aktuell*) conducted by the Robert Koch Institute in 2009 [25]. The results present data on SHS exposure stratified by sex in several age groups (Table 2).

**Table 2:** Exposure to SHS in Germany, stratified by sex and age groups, 2009 ^1^.

Age Groups	People Exposed to SHS (in %)	
	Men	Women
18–29	72.0	61.6
30–39	49.0	27.0
40–49	46.4	28.1
50–59	42.5	24.8
60–69	27.0	17.0
70+	16.2	8.9

^1^ Source: GEDA survey [25].

DYNAMO-HIA aims to use an almost real-life population for modeling purposes. Therefore, the analysis is stratified by sex and age in one-year age categories up to the age of 95 years. The expected number of newborns and a sex ratio of 1.05 are included in the analysis, whereas migration is not. It is a dynamic model using one-year time steps for the reference and intervention scenarios. Explicit risk-factor states are used so that each simulated individual is classified into a specific risk-factor category at every time step. This approach should ensure an accurate and unbiased estimation and increase the transparency of the simulation and resulting output data. DYNAMO-HIA uses a parameter output module to reduce data needs. Therefore, the prevalence of disease is only needed at the population level specified by age and sex and not by every single risk-factor state. In this module, risk factor-specific values are back-calculated using the RR from each risk-factor state on diseases [23].

To compare the effects of policies designed to reduce SHS exposure on future population health, the reference scenario (no change in SHS exposure) is compared with two further scenarios. In these, the transition probabilities between the risk-factor state of no SHS exposure and SHS exposure are changed. In the first scenario, a success rate of 20% in the reduction of SHS exposure is assumed for all age groups. This means that for the percentages of people exposed that are given in Table 2 the exposure will be reduced by 20% for each age group. According to the results of a Cochrane review, the reduction in the percentage of people exposed to SHS varied between 22% and 85% after the implementation of legislative smoking bans. Therefore, a reduction of 20% is quite conservative [5]. Additionally, the second scenario assumes the total eradication of SHS exposure (100% success rate).

The simulation started in the year 2014 and covers 26 years until 2040. Since the risk factor is categorical, the simulation of a small number of individuals as a representative sample of the whole population is enough [21]. Therefore, it was decided to simulate for 10,000 people. The data output was stored and processed in Microsoft Excel Version 2007.

### 2.2. Method of Computation

DYNAMO-HIA uses a “partial micro-simulation” approach for the computation, which is a combination of a macro- and micro-simulation. Usually, in a Markov model, a matrix of transition probabilities is repeatedly applied to a vector of state probabilities [26,27]. In a macro-simulation, a repeated application of the transition matrix is carried out. A model with *n* states, therefore, requires an *n* × *n* transition probability matrix. This approach quickly becomes unfeasible if there is a large number of states. An alternative solution is to implement the Markov model as a stochastic or micro-simulation model. In the micro-simulation, a representative sample of individuals is taken from the state-space, where the probability that an individual with a particular state is in the sample is proportional to the probability of that state. The model simulates the life courses of these individuals from the transition probabilities. Transition rates are used (using a random number generator) to draw the next step for each simulated person (fixed time-step simulation) or a waiting time to the next change of state (discrete event simulation) [21,22].

In DYNAMO-HIA a combination of both approaches is applied in the form of a partial micro-simulation. In this approach, the risk-factor history for each simulated individual is generated similar to a fixed time-step micro-simulation: at each moment in time, each individual has a “risk factor state”, and this state is updated by randomly drawing a new risk-factor state using the transition probabilities between risk-factor states as defined by the transition matrix. The disease part of the model uses a macro-simulation: instead of assigning an individual as affected or not affected by a disease state, the individual is assigned a probability of having the disease [22,23]. The division into a micro- and macro-simulation is done for computational convenience. Both approaches yield the same results when they are used with the same data [28,29].

Since this analysis includes three diseases (IHD, COPD, and stroke), the disease probability aspect consists of a series of probabilities, one for each combination of all the separate disease states. Furthermore, it is important to understand that the risk-factor transition rates are not influenced by the disease states of an individual. After the simulation is run, the probability of a particular disease state can be averaged over all the simulated individuals or over a subgroup of simulated individuals stratified by age and sex [22]. A detailed description of the calculation of diseases is provided by RIVM [27] and Boshuizen *et al.* [22].

## 3. Results

The modeling approach focuses on the estimated impact of SHS exposure for morbidity related to IHD, COPD, and stroke in Germany. The results refer to the calculations of diseases which are estimated as attributable to SHS exposure in 2014 and a projection until 2040, using the reference scenario alongside two further scenarios (20% and 100% reduction in exposure).

### 3.1. Ischaemic Heart Disease

**Figure 1:**
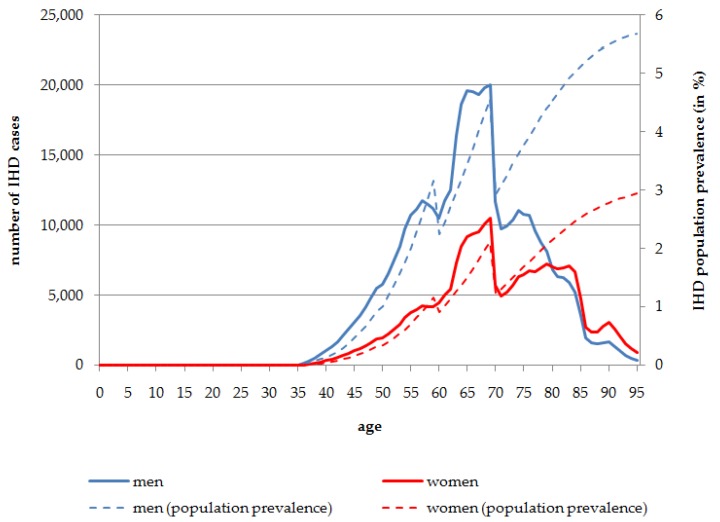
Number of persons affected by IHD attributable to SHS exposure, stratified by age and sex, Germany, 2014.

In Figure 1 the absolute numbers of IHD cases and its population prevalence attributable to SHS exposure stratified by age and sex are presented. According to the results of the reference data used in the modeling approach, the first cases of IHD caused by SHS exposure (129 cases in men and 40 in women) occur at the age of 36 years in the total population living in Germany in 2014. Men are more often affected than women. In the oldest age groups, of people aged 80 years and more, the number of IHD cases attributable to SHS exposure is higher in women, because of the overall larger number of women living to these ages. The peak for the total number of people affected is at the age of 69 years for both men (*n* = 20,018; 4.56% population prevalence at the age of 69 years) and women (*n* = 10,528; 2.11% population prevalence at the age of 69 years). Overall, in the age range 60 to less than 80 years, 268,878 men and 141,276 women are affected by IHD attributable to SHS exposure in 2014. After the peak at the age of 69 years, the total number of IHD cases caused by SHS exposure decreases. Nevertheless, the prevalence of IHD cases caused by SHS exposure increases almost constantly with increasing age. In 2014, 5.68% of men aged 95 years and 2.94% of women aged 95 years of the total population are affected by IHD caused by SHS exposure (Figure 1).

### 3.2. Chronic Obstructive Pulmonary Disease

The number of people affected by COPD is much lower than the IHD prevalence in Germany in 2014. Although COPD occurs more frequently at higher ages, which is comparable to IHD, there are no significant differences either between age groups or between the sexes. According to the health impact assessment, the first cases of COPD occur at the age of 40 years.

**Figure 2:**
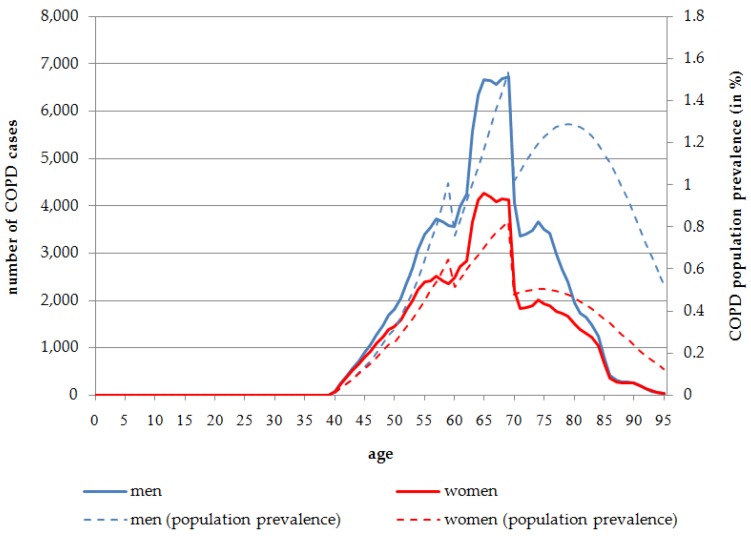
Number of persons affected by COPD attributable to SHS exposure, stratified by age and sex, Germany, 2014.

Overall, 0.35% of the male and 0.22% of the female population are affected by COPD caused by SHS exposure in 2014. In the age group 60 years and older, the population prevalence is much higher with 1.15% for men and 0.55% for women in 2014. The age distribution of COPD cases is almost equal between men and women, although at a slightly higher level for men. At all ages, the absolute number of COPD cases attributable to COPD is higher in men than in women. Overall, 139,103 COPD cases for men, and 92,870 COPD cases for women, are attributable to SHS exposure in 2014. In 2014, more than half of the total COPD cases attributable to SHS exposure occur in the age range of 60 to 75 years (men: *n* = 78,525, 56.45%; women: *n* = 48,335, 52.05%) (Figure 2).

In contrast to the constantly-increasing prevalence rate for IHD, the COPD population prevalence rate declines at the age of 80 years. After reaching a peak at the age of 79 for men (1.29%) and 74 years for women (0.50%), the prevalence rate declines to 0.53% in males and 0.13% in females at the age of 95 years in 2014 (Figure 2).

### 3.3. Stroke

In 2014, overall 1,349,883 stroke cases are expected, indicating a population prevalence of 1.64%. The absolute number of stroke cases is slightly higher in women (*n* = 685,230) than in men (*n* = 664,653). Nevertheless, the population prevalence is almost equal, with 1.63% in women and 1.65% in men. The distribution of stroke cases in terms of the absolute number and population prevalence is reversed due to demographic factors and the larger number of women in the older age groups. Most stroke cases occur in the age range of 60 to 80 years. In people aged 60 years and over, the stroke population prevalence is 6.66% for men and 7.35% for women.

**Figure 3:**
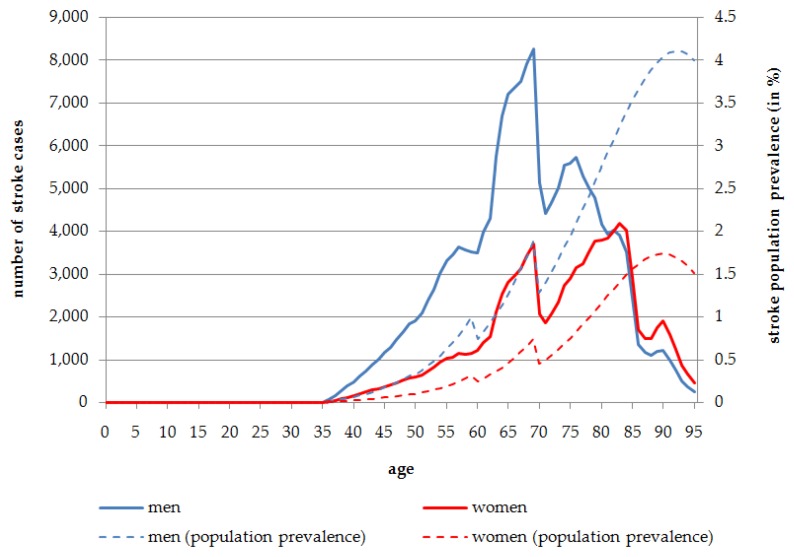
Number of persons affected by stroke attributable to SHS exposure, stratified by age and sex, Germany, 2014.

Exposure to SHS is estimated to be responsible overall for 28.03% of all stroke cases in men (*n* = 186,281) and 14.85% in women (*n* = 101,734) in 2014. Stroke cases attributable to SHS are most frequent in the age range of 65 to 85 years. The highest number of stroke cases caused by SHS exposure is to be found at the age of 69 years in men (*n* = 8258; 1.88% population prevalence) and 83 years in women (*n* = 4195; 1.41% population prevalence) in 2014 (Figure 3). When considering stroke distribution attributable to SHS exposure in the course of aging, Figure 3 shows that before the age of 80 years men are affected more often than women in terms of the absolute number of stroke cases. Although in the population aged more than 80 years, the number of stroke cases is almost equal between men and women, this is due to the fact that more women live to these older ages. 30,921 stroke cases attributable to SHS exposure occur in the age group 80 years and more for men, which indicates a population prevalence of 3.30%. The absolute number of cases for the same age group in women is slightly higher with 35,955 cases leading to a population prevalence of 1.45% (Figure 3).

### 3.4. Projection until 2040

In Table 3 the number of disease cases which are estimated to be attributable to SHS exposure is shown for 2014 and 2040. In 2014, men are assumed to be 1.8 times more likely to suffer from IHD and stroke caused by SHS exposure than women and 1.5 times more likely to be affected by COPD attributable to SHS exposure compared to women. Without any changes in the prevalence of SHS exposure, the number of people affected by IHD caused by SHS exposure is estimated to increase from 441,137 men in 2014 to 525,921 in 2040. The number of disease cases for IHD in women will increase comparably from 246,117 in 2014 to 269,788 in 2040. Under the assumption of no change in the prevalence of SHS exposure, almost 1.5% of the total male population and 0.72% of the female population are estimated to suffer from IHD caused by SHS exposure in 2040.

The disease cases and prevalence rates attributable to SHS exposure are lower for COPD and stroke. Nevertheless, it is estimated that around 231,973 COPD cases and 288,015 stroke cases were attributable to SHS exposure in 2014. The numbers of disease cases indicate an increasing trend under the assumption of no change in SHS exposure. Therefore, a prevalence of 0.3% is estimated each for COPD or stroke cases caused by SHS exposure in 2040. Furthermore, 0.47% of men will be affected by COPD and 0.69% by stroke attributable to SHS exposure.

The two scenarios of SHS prevalence reduction indicate the effects of different policies. For example, the scenario with a 100% success rate—meaning that no more people are exposed to SHS from 2014 onwards—leads to a reduction of 50% compared to the reference scenario in 2040 for all three disease entities. The scenario with a 20% success rate reduces the prevalence rates for the three diseases in 2040 to a level which is comparable to the situation in 2014 (Table 3).

**Table 3:** Disease cases and population prevalences attributable to SHS exposure, Germany, 2014/2040.

Scenarios	IHD		COPD		Stroke	
	Men n (%)	Women n (%)	Men n (%)	Women n (%)	Men n (%)	Women n (%)
**2014**						
Reference scenario	441,137 (1.09)	246,117 (0.58)	139,103 (0.35)	92,870 (0.22)	186,281 (0.46)	101,734 (0.24)
**2040**						
Reference scenario	525,921 (1.46)	269,788 (0.72)	170,525 (0.47)	99,863 (0.27)	248,792 (0.69)	112,438 (0.30)
Scenario 20% success rate	420,737 (1.16)	215,831 (0.58)	136,420 (0.38)	97,914 (0.21)	199,033 (0.55)	89,950 (0.24)
Scenario 100% success rate	262,960 (0.73)	134,894 (0.36)	85,262 (0.24)	49,946 (0.13)	124,396 (0.34)	56,219 (0.15)

## 4. Discussion

The study focused on disease entities which show their impact mainly in the elderly, because the effect of IHD, COPD, and stroke is mainly due to their character as chronic diseases. Although both IHD and stroke also impact upon mortality by premature deaths which may be caused by acute events, SHS exposure and its effects primarily influence morbidity. The study results indicate that it is mostly people aged 60 years and more who are affected by the impact of SHS exposure. Due to the higher population prevalence of these diseases in the German population, IHD accounted mainly to the disease burden attributable to SHS exposure. About 440,000 men and 250,000 women were estimated to suffer from IHD caused by SHS exposure in Germany in 2014. The absolute number of stroke cases attributable to SHS was also higher compared to COPD cases (Table 3). Although the effect sizes for the associations between SHS exposure and adverse health effects applied in this HIA were higher in women than in men for all three outcomes, the absolute number of cases attributable to SHS is lower in women than in men. This is due to the higher population prevalence rates of the three diseases in men compared women.

The need to implement new policies is apparent due to the rising number of disease cases attributable to SHS exposure. Under the assumption that the population prevalences of these diseases and the prevalence of SHS exposure remain constant, the total number of cases will increase due to demographic aging. According to the estimations, 795,709 IHD cases (+15.8%), 270,388 COPD cases (+16.6%), and 361,230 stroke cases (+25.4%) are expected for 2040. The calculations for the two scenarios assuming a 20% decrease in the prevalence of SHS exposure and a total eradication of SHS exposure indicate the expected impact of these policies on the population’s health. According to this calculation, the scenario with a 20% decrease in SHS exposure will lead to a number of cases attributable to SHS exposure that is nearly on the same level in 2040 as in 2014. A total eradication of SHS exposure will make it possible that the number of cases falls to just under half of the number in 2040 compared to 2014. Although the total numbers of cases and the effects are quite large, the impact of policies aiming to protect people from adverse health effects on the total population prevalence for all three disease entities seems to be comparatively low. This can be explained by the fact that chronic diseases develop over a long time period. Therefore, the effects of exposure may appear after several years or even decades of exposure. Furthermore, it has to be considered that SHS exposure is just one of several risk factors, such as active smoking, dietary behavior, physical activity, genetics, *etc.*, contributing to the development of these chronic conditions [30].

The current primary preventive task, with respect to morbidity and mortality in the German population due to tobacco use and SHS exposure, is the implementation of strategies for the cessation of active smoking and the prevention of smoking initiation in adolescents. Additionally, an SHS exposure prevention is required to protect non-smokers from adverse health effects. This can be substantially supported by public smoking bans [6]. The effects of SHS exposure reduction or elimination are well documented [5]. Nevertheless, much of SHS exposure happens at home. Therefore, further strategies are needed to reduce the exposure also in the familiar setting and not only in public places.

It is estimated that, through the effective implementation of a comprehensive workplace smoking ban, 4%–5% of coronary heart disease cases, 13%–16% of asthma bronchiale cases, 7%–8% of COPD cases, 15%–18% of pneumonia cases, 4% of lung cancer cases and 11%–13% of stroke cases could be avoided [31]. These estimates are generally consistent with the calculations in this study. They provide additional information due to the reduction of cases being given in percentages. The calculations provided in this study refer to absolute numbers of people being affected by IHD, COPD, or stroke attributable to SHS exposure. Furthermore, this information includes changes in the demographic structure of the total population, accounting for demographic aging. For this reason, the absolute number of disease cases attributable to SHS exposure increases during the observed time period in the reference scenario assuming no change in SHS exposure. The scenarios provide estimates for the reduction or elimination of SHS exposure respectively, in the total population. Therefore, these estimates do not account for changes in SHS exposure in different sub-groups. The estimates for the effects of different scenarios in this study are the scientific basis for the implementation of policies that will provide substantial improvements in a population’s health [32].

### Limitations

The results face several limitations because a number of assumptions and uncertainties lie behind the figures presented for the impact of SHS exposure on populations’ health and the effects of different scenarios. First of all, it was assumed that the effect sizes differ between sexes but not in different age groups. This assumption was made because nearly all studies provide estimates of effect sizes which are not stratified for age. In particular, children and people in older age groups might be more susceptible to the adverse health effects. Unfortunately, the analysis could not account for vulnerability in different age groups due to lack of data. Nevertheless, the impact of SHS exposure on diseases in different age groups was accounted for due to the disease prevalences and exposure prevalences. In addition, the data on RR were derived from cohort studies but also from case-control studies. For case-control studies the RR were derived from the provided odds ratios [24]. This major limitations indicate that the results should not be interpreted as precise estimates but rather, as a first approximation to quantifying the effects of SHS exposure, considering the age-structure of the population.

However, the approach used for the HIA combines the best of existing model approaches with the aim of constructing an efficient Markov model-based disease model that was implemented in the DYNAMO-HIA model. The limitations of the modeling approach are described in detail elsewhere [22,33,34]. In our study, we did not consider competing risk factors, such as smoking, further environmental risk factors, or beneficial factors, such as better medication. The projection of future disease prevalences is relatively rare in chronic disease modeling, because most Markov models project only incidence and mortality. Nevertheless, the projections of disease prevalences provide important information which can be used later on for the calculation of summary measures of population health such as DALYs [22]. In this study, the projection of disease prevalences attributable to SHS exposure was the focus. A burden of disease assessment can be done in a next step, by including further information on age-stratified effect sizes for the association between SHS exposure and adverse health effects.

For the effects of the scenarios, two assumptions were made. The first one is a total eradication of SHS exposure and the second one is a reduction of 20% in exposure. The second one is also a conservative assumption which should be compared to the maximum effects. In this context, a further limitation for interpreting the results is the absence of data on historical trends for disease incidence and prevalence, which is necessary to predict future trends in the absence of interventions [33]. However, this is the most suitable approach to calculate the effects of SHS exposure by incorporating information into an already existing tool that contains data on population structure and diseases. The impact of SHS exposure may differ among federal states in Germany, because the SHS exposure prevalences may be different due to variations in legislation. Overall, the HIA faces some limitations in the calculations which are mainly forced by the uncertainties due to the various assumptions.

## 5. Conclusions

The uncertainties indicate the need for more research on SHS exposure and its effects, as well as research on the impact of tobacco control strategies. So far, the main focus has been the effects of reductions in hospital admissions for acute myocardial infarction after the introduction of smoke-free legislation. However, evidence for the impact of anti-smoking legislation on other cardiovascular or respiratory outcomes among non-smokers is still lacking [35]. To address this research need, this study was conducted. It is the first study which calculated and projected the impact of IHD, COPD, and stroke attributable to SHS exposure for the German population using a dynamic modeling approach for the HIA. The results highlight the relevance of SHS exposure, because it affects several chronic disease conditions and has a major impact on the population’s health. Although several acts to achieve smoke-free work-places and public places have already been passed, more efforts are still needed to protect especially vulnerable populations, such as children. Generally, non-smokers are involuntarily exposed to a risky behavior caused by others. Therefore, non-smokers, as well as smokers, must be addressed in various settings to reduce SHS exposure. This is needed to reduce SHS exposure substantially and, therefore, protect non-smokers from its adverse health effects.

## Abbreviations

The following abbreviations are used in this manuscript:

COPD: Chronic obstructive pulmonary disease
HIA: Health Impact Assessment
IARC: International Agency for Research on Cancer
IHD: Ischaemic heart disease
RR: Relative risk
SHS: Second-hand smoke
YPLL: Years of Potential Life Lost
